# Gait data from 51 healthy participants with motion capture, inertial measurement units, and computer vision

**DOI:** 10.1016/j.dib.2024.110841

**Published:** 2024-08-14

**Authors:** Jere Lavikainen, Paavo Vartiainen, Lauri Stenroth, Pasi A. Karjalainen, Rami K. Korhonen, Mimmi K. Liukkonen, Mika E. Mononen

**Affiliations:** a Department of Technical Physics, University of Eastern Finland, P.O. Box 1627, Yliopistonranta 8 (Melania building), 70211 Kuopio, Finland; b Diagnostic Imaging Centre, Kuopio University Hospital, Wellbeing Services County of North Savo, Puijonlaaksontie 2, 70210 Kuopio, Finland

**Keywords:** Gait data, Inertial measurement, Video capture, Gait analysis, Openpose

## Abstract

We present a dataset comprising motion capture, inertial measurement unit data, and sagittal-plane video data from walking at three different instructed speeds (slow, comfortable, fast). The dataset contains 51 healthy participants with approximately 60 walking trials from each participant.

Each walking trial contains data from motion capture, inertial measurement units, and computer vision. Motion capture data comprises ground reaction forces and moments from floor-embedded force plates and the 3D trajectories of subject-worn motion capture markers. Inertial measurement unit data comprises 3D accelerometer readings and 3D orientations from the lower limbs and pelvis. Computer vision data comprises 2D keypoint trajectories detected using the OpenPose human pose estimation algorithm from sagittal-plane video of the walking trial. Additionally, the dataset contains participant demographic and anthropometric information such as mass, height, sex, age, lower limb dimensions, and knee intercondylar distance measured from magnetic resonance images.

The dataset can be used in musculoskeletal modelling and simulation to calculate kinematics and kinetics of motion and to compare data between motion capture, inertial measurement, and video capture.

Specifications Table

**Table:** 

Subject	Sport Sciences, Therapy and Medicine
Specific subject area	Biomechanical motion analysis and musculoskeletal simulation and modelling of human locomotion.
Type of data	Table: demographic and anthropometric variables of subjects in XLSX format Time Series: ground reaction forces and moments from force plate data in C3D and Vicon-compatible formats; 3D trajectories of motion capture markers in C3D and Vicon-compatible formats; 2D trajectories of keypoints identified from video data using a human pose estimation algorithm in JSON format; accelerometer and orientation data from inertial measurement units in Xsens- and MATLAB-compatible formats Raw: ground reaction forces and moments; motion capture marker trajectories; keypoint trajectories; accelerometer data from inertial measurement units Filtered: orientation data of inertial measurement units, generated from the raw data of the inertial measurement units with a proprietary Kalman filter (see [1] for details) Analyzed: intercondylar distances from magnetic resonance images (provided in the demographic and anthropometric variables table)
Data collection	The motion data was collected using motion laboratory equipment (cameras and 42 reflective markers at 100 Hz, a video camera aimed at the sagittal plane of motion at 100 Hz, force plates at 1000 Hz), and wearable motion sensors (inertial measurement units). We utilized the Vicon Nexus motion capture software for collecting motion capture data and Xsens MT Software Suite 4.6 for collecting inertial measurement unit data at 100 Hz. The OpenPose human estimation algorithm was used to detect 2D keypoints from planar video data. Demographic and anthropometric data were collected with measuring tape, calipers, a survey, and low-field magnetic resonance imaging equipment.
Data source location	The data was collected in the HUMEA motion laboratory (https://sites.uef.fi/humea/) in the Department of Technical Physics, University of Eastern Finland, Kuopio, Finland.
Data accessibility	Repository name: Zenodo Data identification number: 10.5281/zenodo.10559503 Direct URL to data: https://zenodo.org/records/10559504 Instructions for accessing these data: Freely downloadable from Zenodo [2]. The description in Zenodo has more detailed information about the data. Accessing extracted IMU data requires the MATLAB software.

## 1 Value of the Data

• The data is useful because it provides an extensive motion capture dataset with marker trajectories and ground reaction forces, as well as wearable sensor data from inertial measurement units and keypoint trajectories identified from sagittal-plane video data using machine vision.
• Researchers working in human motion analysis can use the data to conduct their own research in human gait analysis and comparison and validation of motion capture techniques.
• The data can be used for quantifying repeatability of kinematic and kinetic parameters over identically instructed walking trials, evaluating the effect of walking speed on kinematic and kinetic parameters, validating portable measurement techniques against the gold standard method, and comparing wearable sensor data to video data extracted with machine vision.
• The number of subjects, walking trials per subject, and the variety of walking speeds during the trials enable between- and within-subject gait analysis and comparison of various gait parameters at different speeds.
• The optical marker trajectories and force plate data are accompanied by acceleration and orientation data from wearable inertial measurement units and keypoint trajectories identified from video data using the OpenPose [3] algorithm.
• The provided demographic information, manual measures of the participants’ lower extremities, and intercondylar distances of the knee calculated from magnetic resonance images may provide additional useful information for motion analysis studies in future, for example to create a gait classification model based on machine learning approaches.

## 2 Background

The number of openly available and extensive 3D gait datasets is limited [4]. Access to gait data is important because walking is the primary form of human locomotion and mechanics associated with it are linked to many diseases [5]. The increasing prevalence of data-driven machine learning methods further emphasizes this need for data [6]. Portable modalities have been investigated as one way to enable accessible biomechanical assessment of human motion [7,8], so their inclusion in addition to standard motion capture data would increase the value of gait datasets in general.

We followed standard procedures and techniques while recording the data to produce a dataset that can be used in musculoskeletal analysis of human biomechanics. The dataset contains the required data to conduct these analyses using motion capture data (i.e., force plate data and marker trajectories), and additionally, data from two portable alternative modalities (inertial measurement units and human pose estimation from video data). The dataset can be used, e.g., to estimate knee or hip joint loading with different gait speeds; the inclusion of portable modalities enables comparison of biomechanical outcomes between standard motion laboratory-grade measurements and portable measurements to, e.g., evaluate how well portable modalities match with the reference method. The data could also be used to classify gait according to its biomechanical characteristics.

## 3 Data Description

**Fig. 1:**
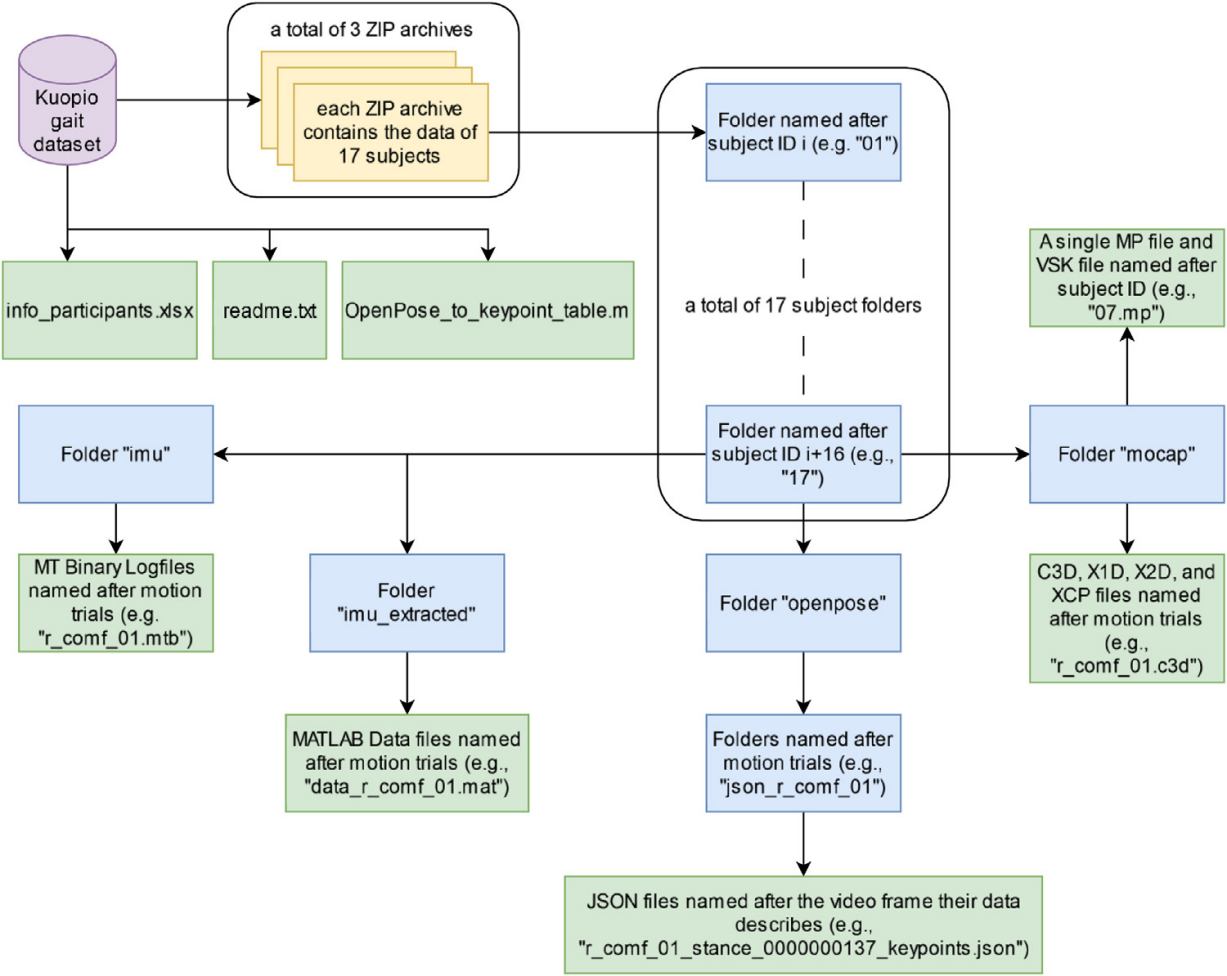
Structure of the Kuopio gait dataset. Folders are colored blue and files green.(For interpretation of the references to color in this figure legend, the reader is referred to the web version of this article.)

The data was collected in the HUMEA motion laboratory, Department of Technical Physics, University of Eastern Finland, Kuopio, Finland (https://sites.uef.fi/humea/humea-laboratory/). The data collection started in April 2022 and ended in July 2023. The aim of the data collection was to collect a dataset of healthy gait reference motion capture data and data from two portable modalities (inertial measurement units and video camera) such that the feasibility of using data from portable modalities could be evaluated against the reference motion capture data. The reference motion capture data comprised the 3D trajectories of motion capture markers and the ground reaction forces and moments from floor-embedded force plates. The portable modalities were inertial measurement units (IMU), which are wearable motion sensors, and video camera data. The video camera data was further analyzed to retrieve the 2D trajectories of keypoints, which are locations on the human body (e.g., joints and prominent anatomical locations) identified from video data using the OpenPose human pose estimation algorithm. Fig. 1 shows the structure of the collected dataset. All data described here was collected by the authors.

On the top level, the dataset contains a readme (readme.txt), an Excel table containing information about the participants (info_participants.xlsx), a MATLAB (version R2023a) script (OpenPose_to_keypoint_table.m), and three ZIP archives (measurement_data_1_to_17.zip, measurement_data_18_to_34.zip, measurement_data_35_to_51.zip).

The Excel table contains the demographic and anthropometric information of each of the 51 subjects. The demographic information includes the subject's body mass, height, age, and sex. The anthropometric information contains measurements of body dimensions of the subject. These body dimensions include manual measurements of the widths of knees and ankles (left knee width, LKW; right knee width, RKW; left ankle width, LAW; right ankle width, RAW), the lengths of thighs and shanks (left thigh length, LTL; right thigh length, RTL; left shank length, LSL; right shank length, RSL), and the distance between the anterior superior iliac spines (ASIS) of the subject (inter-ASIS distance, IAD). Additionally, the body dimensions data contains knee widths calculated from motion capture markers and intercondylar distances of the knee analyzed from magnetic resonance images. Finally, the table also contains the subject's self-identified dominant leg. The exact descriptions of the columns and the units in the Excel table are described in the readme file. Tables 1 and 2 summarize the demographic and anthropometric data in the Excel table.

**Table 1:** Demographic summary of the participants (*N* = 51) in the dataset.

	Mass [kg]	Height [cm]	Age [years]
Mean	77.0	174.6	29.7
Standard deviation	14.2	7.0	7.9
Range	54–136	161–189	20–68

**Table 2:** Summary of lower body dimensions of the participants in the dataset. The variables are the distance between the anterior superior iliac spines (ASIS), denoted here as inter-ASIS distance (IAD); left knee width (LKW), right knee width (RKW), left ankle width (LAW), right ankle width (RAW), left thigh length (LTL), right thigh length (RTL), left shank length (LSL), and right shank length (RSL). All values are in millimeters.

	IAD	LKW	RKW	LAW	RAW	LTL	RTL	LSL	RSL
Mean	236.8	98.6	99.4	69.3	68.7	402.0	400.1	420.0	423.8
Standard deviation	17.2	5.6	6.1	5.3	5.1	25.5	29.2	25.5	26.1
Range	193–285	86–113	85–121	60–78	61–78	350–449	333–468	375–498	370–509

The ZIP archives contain folders for each participant named in 2-digit format after the ID of the participants (e.g., “05” for the participant with ID 05). Within each participant folder, there are four subfolders: “imu”, “imu_extracted”, “mocap”, and “openpose”.

The folder “imu” contains .mtb files exported from the Xsens MT Manager 4.6 software; there .mtb files contain the orientation and acceleration data from inertial measurement units. Each .mtb file contains the time series of orientation and acceleration of a single walking trial from all seven IMUs. The folder “imu_extracted” contains the same data, but saved into MATLAB structs using the Xsens Device API (legacy version that comes with MT Software Suite 4.6 for MTw Awinda sensors). Unless you wish to use the Xsens Device API to customize the IMU data extraction process, you are encouraged to use the files in “imu_extracted” and ignore the files in “imu”.

The folder “mocap” contains the C3D, X1D, X3D, and XCP files for each walking trial, generated by the Vicon Nexus (version 2.12) motion capture software. C3D is a standard format for processed motion capture data, while X1D, X2D, and XCP are Vicon's own formats containing unprocessed analog data, unprocessed 2D marker data detected by Vicon infrared cameras, and calibration data of cameras, respectively. In the case of our dataset, the analog data contains the ground reaction forces and moments of force plates. Each subject also has a VSK and MP file. The VSK file is a Vicon skeleton file containing the marker arrangement and connections, while the MP file contains subject and model specific parameters; both are used by Vicon Nexus software. If you do not use Vicon products, you can access the data using only the C3D files.

The folder “openpose” contains subfolders for the JSON files of keypoint trajectories, with each JSON file containing the keypoint positions for a single frame of video data. Hence, each subfolder in the “openpose” folder contains the data for a single walking trial, split per frame into multiple JSON files. Each JSON file contains the 2D coordinates and confidence of detection (between 0 and 1) of all detected keypoints in that frame of video data.

The files and folders in the four subfolders are organized by different trials. There are separate files for each walking trial in subfolders “imu” and “imu_extracted”, separate folders for each walking trial in “openpose”, and several files for each walking trial in “mocap”. All files and folders are named after the walking trial, e.g., “r_comf_02” to make separating the data of different trials simple. The naming convention is further explained in Table 3 of the methods section.

**Table 3:** Configurations of the walking trials. Six configurations were used, formulated by walking speed (comfortable, slow, fast) and the side of the participant that was visible to the video camera placed to capture sagittal-plane motion (left or right). The recorded files are named accordingly, e.g. (“r_comf_02” for the second walking trial at comfortable speed where the right side of the participant was facing the camera).

Configuration	Speed	Visible side to sagittally placed video camera	File descriptor
1	Comfortable	Right	r_comf_XX
2	Slow	Right	r_slow_XX
3	Fast	Right	r_fast_XX
4	Comfortable	Left	l_comf_XX
5	Slow	Left	l_slow_XX
6	Fast	Left	l_fast_XX

The MATLAB script “OpenPose_to_keypoint_table.m” contains a function for reading keypoint trajectories from JSON files into MATLAB tables, where they are easier to analyze provided you have access to MATLAB.

## 4 Experimental Design, Materials and Methods

The data was collected from 51 willing participants during two measurement visits: 1) walking measurements in a motion laboratory and 2) a magnetic resonance imaging session where each participant's knee was imaged.

In the beginning of the walking measurements but before recording actual walking trials, each participant's mass, height, age, gender (33 male, 18 female), dominant leg (49 right, 2 left), and lower body dimensions were collected with manual measurements (force plate for mass, measuring tape and caliper for length measures) and a survey. The lower body dimensions included the distance between the anterior superior iliac spines (ASIS; the distance between them is also known as the inter-ASIS distance), widths of knees and ankles, and lengths of thighs and shanks. The knee widths were measured from the skin surrounding the medial epicondyle to the lateral epicondyle (while compressing soft tissue lightly with the caliper), ankle widths from the medial malleolus to the lateral malleolus, thigh lengths from the greater trochanter of the femur to the lateral epicondyle of the knee, and shank lengths from the lateral epicondyle of the knee to the lateral malleolus of the ankle. Anatomical landmarks were identified with palpation.

The walking measurements were conducted in the HUMEA motion laboratory in the Department of Technical Physics, University of Eastern Finland, Kuopio, Finland. Motion capture data was collected using three floor-embedded force plates, 10 Vicon Vero cameras, a Vicon Vue video camera, 42 reflective markers placed on the participants, and Vicon Nexus software (Vicon Motion Systems Ltd, UK). The software recorded reconstructed 3D trajectories of the reflective markers, ground reaction forces and moments and centers of pressure from force plates, and video of walking. The video camera was used to capture the motion of the subject on the sagittal plane, while the other equipment listed in this paragraph are part of a standard optical motion capture protocol.

Individual reflective markers were placed on the participant's skin and clothing on left and right shoulder acromia, on the manubrium of the sternum, on the 7th cervical vertebra, on both epicondyles of the knees, on both malleoli of the ankles, behind the heels, and on the 1st distal and 4th proximal phalanges. Furthermore, two markers were placed along anteroposterior lines on the IMUs on both feet. The participant was equipped with marker clusters behind the pelvis, on both thighs and on both shanks; these clusters had four markers each placed asymmetrically.

In addition to optical motion capture and its preparation (the placing of reflective markers on the participant), the participant was also outfitted with seven Xsens MTw Awinda (Movella Inc, Henderson, NV, USA) wireless inertial measurement units (IMUs): behind the pelvis, on both thighs, on both shanks, and on both feet (taped above the metatarsals). The pelvis and thigh IMUs were fitted into a slot on the marker clusters, but the IMUs on the shank were strapped below the marker clusters. The IMUs estimated their orientations from accelerometer, gyroscope, and magnetometer data using the proprietary Xsens Kalman filter sensor fusion algorithm. The orientations were described with respect to coordinate axes that were determined using accelerometer and magnetometer data.

The IMUs also utilized accelerometer and magnetometer data for determining their coordinate axes. The IMUs then described their orientation with respect to those coordinate axes.

After preparing the motion laboratory and the participant, the participant was ready to be measured with three separate modalities that would be used concurrently during the walking measurements: 1) optical motion capture, 2) inertial measurement units, and 3) video capture on the sagittal plane. While optical motion capture represented the standard method that is commonly used in studies of human motion, inertial measurement units and video capture represented more portable but possibly less accurate methods of collecting motion data.

During the walking measurements, the participants were instructed to walk with six sets of configurations comprising permutations of three walking speeds and two walking directions (Table 3). The walking speeds were self-selected comfortable speed, slow speed (instructed as 25 % slower than comfortable) and fast speed (instructed as 25 % faster than comfortable). Approximately ten successful walking trials were conducted and recorded per unique instruction, resulting in a total of 60 walking trials for each participant.

**Fig. 2:**
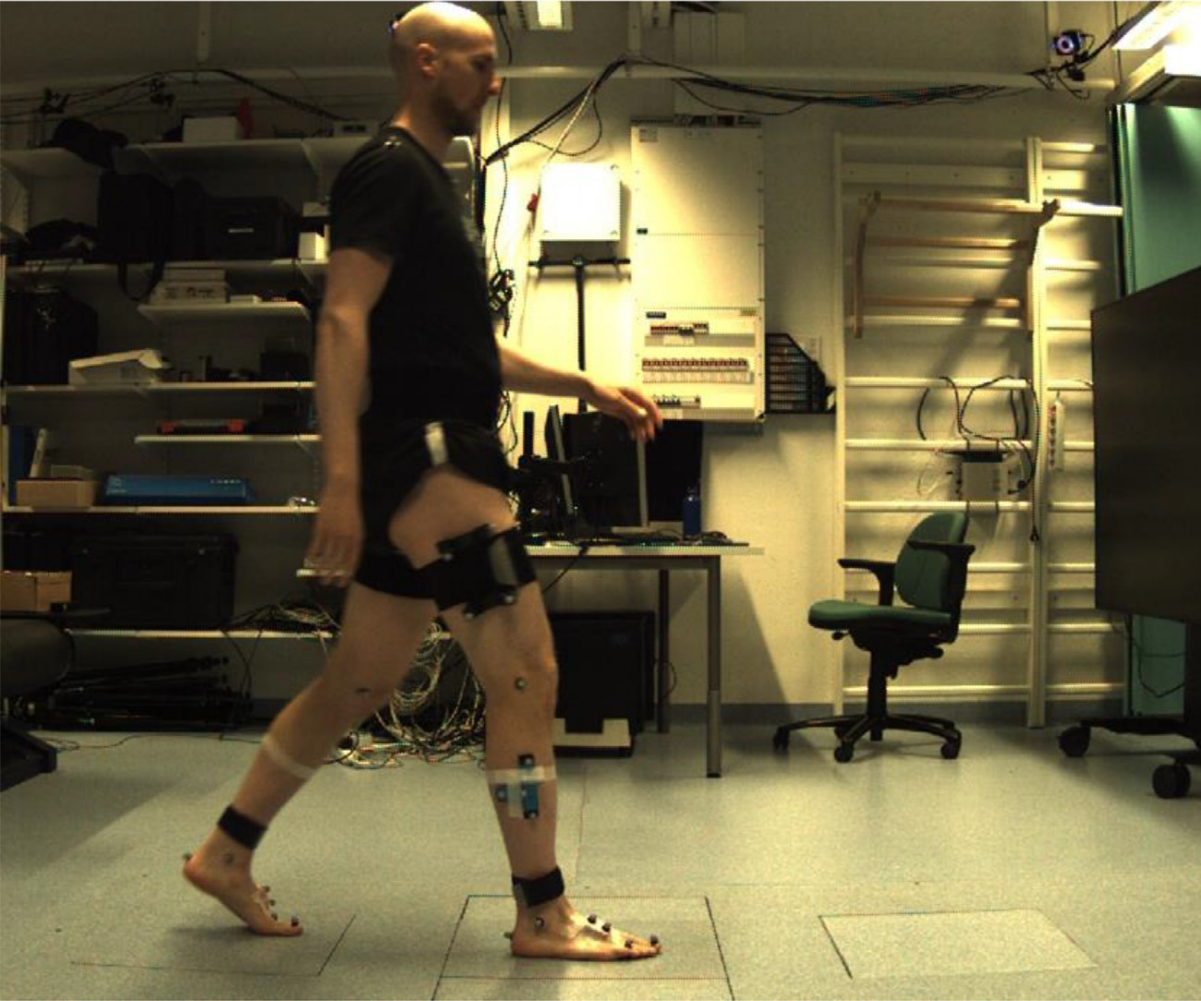
A single frame from the video camera capturing the walking trials on the sagittal plane. Rectangular sections in the floor indicate the floor-embedded force plates. The participant is walking with his right side facing the camera and has stepped on the middle force plate with his right leg. Marker clusters and individual markers are visible on the participant's legs. In the upper right corner, one of the Vicon infrared cameras tracking the markers is visible.

**Fig. 3:**
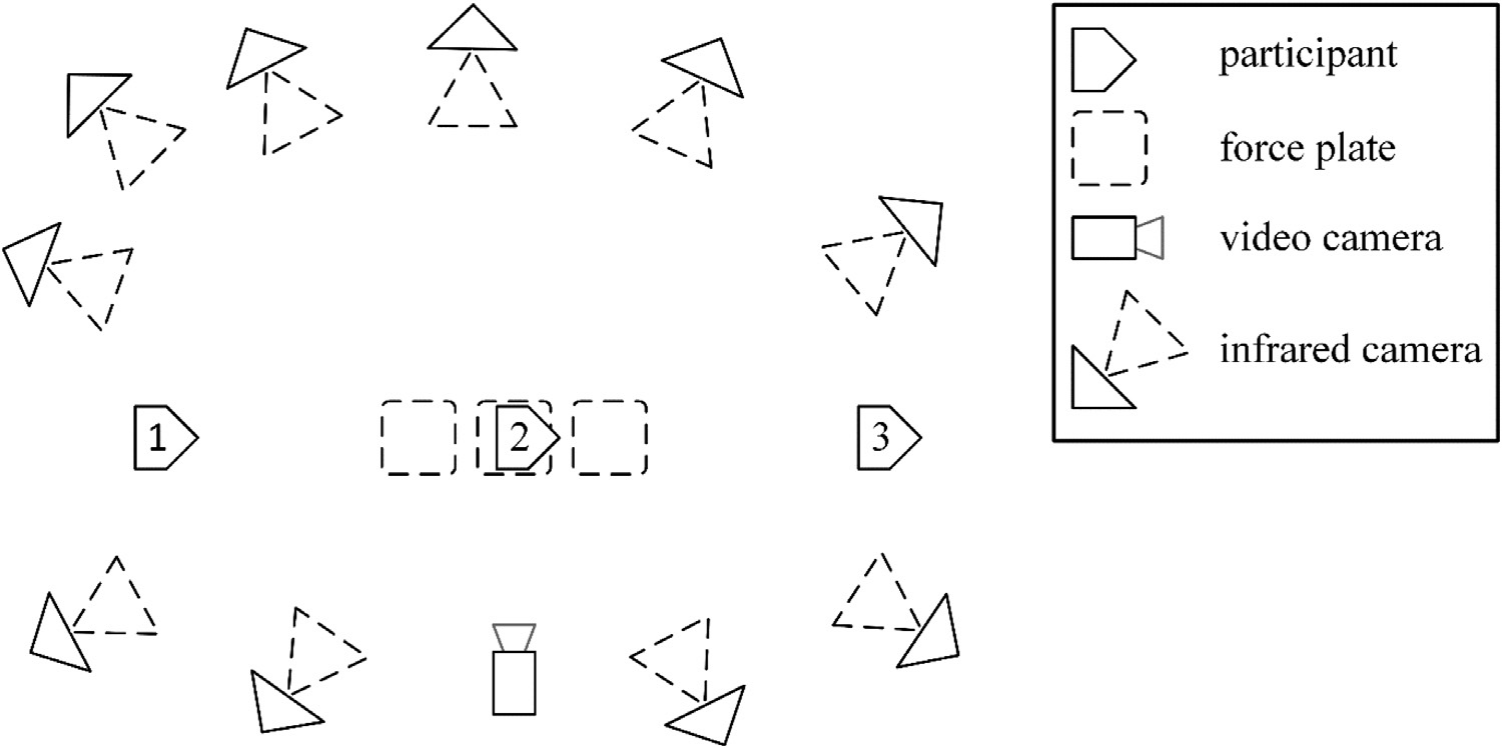
An illustration of the walking trials in the motion laboratory. Each walking trial started with the participant standing still and facing the walking direction (1). After accelerating to an approximately constant walking speed, the participant walked over three floor-embedded force plates, stepping at least on the middle one (2). After walking over the force plates, the walking trial ended once the participant stopped and stood in a similar pose as before starting the motion, still facing the walking direction (3). The participant was equipped with reflective markers and inertial measurement units (not illustrated). The infrared cameras tracked the positions of the markers, the force plates collected the ground reaction forces and moments between the participant's feet and the ground, the video camera recorded a video of the motion in the sagittal plane, and the inertial measurement units measured their own acceleration and orientation during the walking trial.

The walking trials started when the participant was standing still in a neutral position. They were allowed to practice and adjust their starting position so that they could step on the middlemost of the three force plates with their dominant leg and had enough distance to accelerate to a constant speed by the time they stepped on the force plate (Fig. 2). After that, they decelerated and stopped to another static standing trial, facing the same way as they did during walking (Fig. 3).

The motion capture software automatically synchronized marker, force plate, and video data. However, IMU data had to be synchronized with an analog voltage signal from the MT Software Suite 4.6 controlling the IMUs whenever the IMU measurement started or ended. The motion capture software was set to start and stop capturing when it detected the voltage signal from MT Software Suite, thus synchronizing the IMUs to the other data.

In addition to the walking trials, the motion capture data includes static standing trials and three kinds of functional calibration trials: squatting, knee extension, and hip rotation (star arc movement) for use with SARA [9] and SCoRE [10] functional calibration protocols, respectively. These additional motion trials can be used, e.g., to determine joint centers or lengths of body segments when conducting biomechanical analysis of human motion. Therefore, they complement the data from the walking trials.

**Fig. 4:**
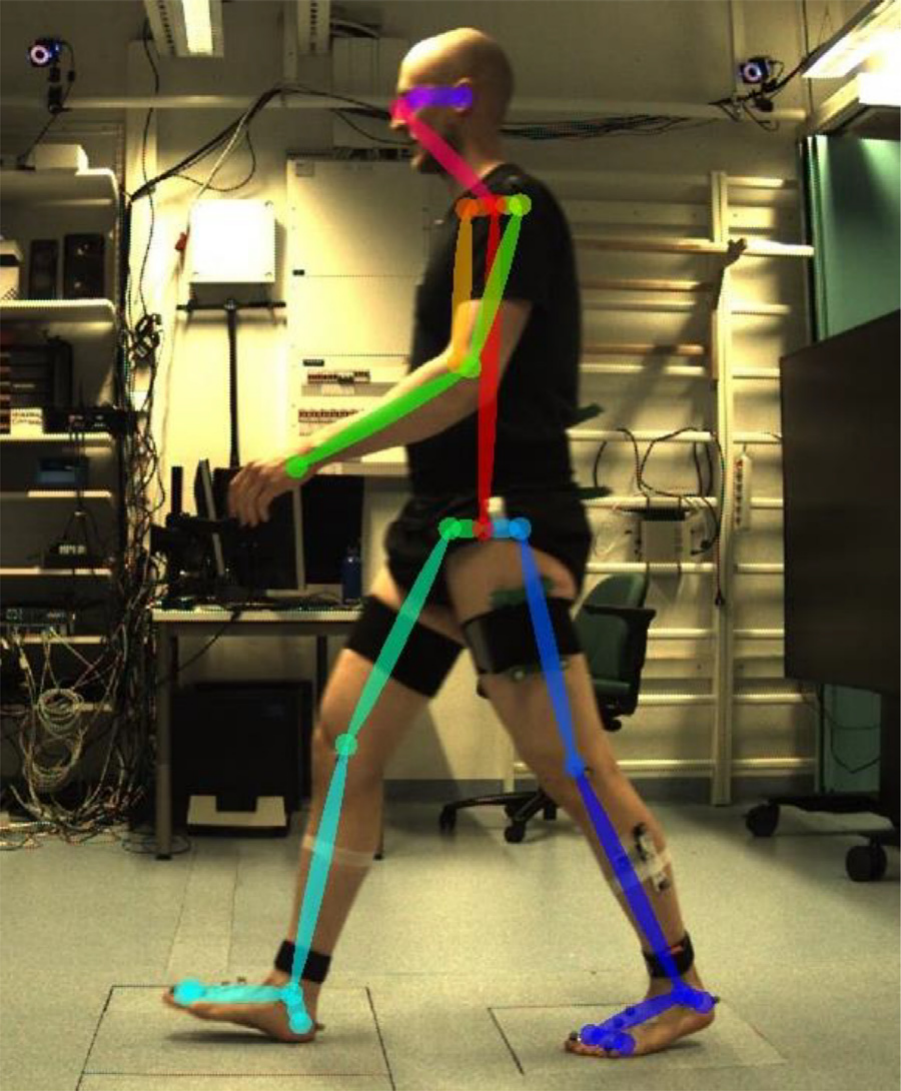
A single frame from the video camera with keypoints detected using OpenPose overlaid on the image.

The motion capture files and IMU files contain marker trajectories at 100 Hz and force plate data at 1000 Hz from complete walking trials, but no video data. The sagittal-plane video data recorded at 1280×720 resolution and 100 Hz was separately analyzed with the human pose estimation algorithm OpenPose [3] using a resolution of 480×272. We used the BODY_25 model to detect the trajectories of 25 keypoints from the video data during the stance phase only (i.e., when the middle force plate was stepped on; see Fig. 4). These keypoint trajectories are available in the “openpose” folder.

Finally, during a separate session, the knee of the participant's dominant leg was scanned with low-field magnetic resonance imaging. The magnetic resonance images are not provided but the dataset includes the knee intercondylar distances of the dominant leg calculated from the images.

## Limitations

• The keypoint trajectories were detected from video frames that sometimes had the operator of the equipment in the background and may thus contain artefact in the keypoint trajectories when the human pose estimation algorithm fails to differentiate between the participant and the operator.
• Some motion capture files contain trials where the participant failed to step on the middle force plate as instructed, resulting in erroneous ground reaction forces, moments, and centers of pressure. We kept these files in the database because they may still be useful for kinematic studies.
• The participants are mostly young people recruited in a university campus, so their gait may not be representative of the population at large.
• We do not provide a comparative analysis of errors between standard motion capture and portable modalities. The reader is referred to, e.g., [[11], [12], [13]] for orientation errors of inertial measurement units, and [[14], [15], [16]] for kinematics errors utilizing OpenPose for keypoint detection.

## Ethics Statement

The study and data collection were reviewed and approved by the University of Eastern Finland Committee on Research Ethics (statement no 16/2022). All participants were volunteers who gave their informed consent to participate. The measurements were performed in line with the principles of the Declaration of Helsinki.

## CRediT authorship contribution statement

**Jere Lavikainen:** Conceptualization, Methodology, Software, Formal analysis, Investigation, Resources, Data curation, Writing – original draft, Visualization. **Paavo Vartiainen:** Conceptualization, Methodology, Investigation, Resources, Data curation, Writing – review & editing, Supervision. **Lauri Stenroth:** Conceptualization, Methodology, Software, Validation, Resources, Data curation, Writing – review & editing, Supervision. **Pasi A. Karjalainen:** Resources, Writing – review & editing, Supervision, Funding acquisition. **Rami K. Korhonen:** Writing – review & editing, Supervision, Funding acquisition. **Mimmi K. Liukkonen:** Resources, Writing – review & editing, Funding acquisition. **Mika E. Mononen:** Conceptualization, Resources, Data curation, Writing – review & editing, Supervision, Project administration, Funding acquisition.

## Data Availability

Kuopio gait dataset: motion capture, inertial measurement and video-based sagittal-plane keypoint data from walking trials (Original data) (Zenodo)
